# Detection, prevention and treatment of COVID‐19 and opportunities for nanobiotechnology

**DOI:** 10.1002/VIW.20200181

**Published:** 2022-03-15

**Authors:** Qing Bao, Tao Yang, Mingying Yang, Chuanbin Mao

**Affiliations:** ^1^ School of Materials Science and Engineering Zhejiang University Hangzhou Zhejiang China; ^2^ College of Animal Science Institute of Applied Bioresource Research Zhejiang University Hangzhou Zhejiang China; ^3^ Department of Chemistry and Biochemistry University of Oklahoma Norman Oklahoma USA

**Keywords:** diagnosis, nanobiotechnology, nanotechnology, prevention, treatment, virus

## Abstract

Since the outbreak of COVID‐19, the number of confirmed cases and deaths has increased globally at a dramatic speed. In view of the serious health threat to humans, this review discusses the state‐of‐the‐art studies about fighting this disease. It summarizes the current strategies and recent advances in detecting, preventing, and treating COVID‐19 and interprets the underlying mechanisms in detail. Detection of COVID‐19 can be successfully achieved by multiple techniques such as polymerase chain reaction, computed tomography imaging, and nano‐biosensing. Inactivated virus vaccine, nucleic acid vaccine, and different nanoparticles have been employed to effectively prevent COVID‐19. A variety of agents such as antiviral agents, neutralizing antibodies, and nanotherapeutics have been developed to treat COVID‐19 with exciting efficacy. Although nanobiotechnology has shown great potential in the diagnosis, prevention, and treatment of COVID‐19, efforts should be made to explore new biocompatible nano‐biomaterials to advance this field to clinical applications. Hence, nanobiotechnology paves a new way to detect, prevent, and treat COVID‐19 effectively.

## Graphical abstract

Since the outbreak of COVD‐19, the number of confirmed cases and deaths has increased globally at a dramatic speed. In view of the serious health threat to humans, this review discusses the state‐of‐the‐art studies about fighting this disease. It summarizes the current strategies and recent advances in detecting, preventing, and treating COVID‐19 and interprets the underlying mechanisms in detail.

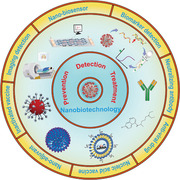

## 1 INTRODUCTION

There is a serious health threat to humans since the outbreak of severe acute respiratory syndrome coronavirus 2 (SARS‐CoV‐2) in December 2019. This virus is the causative agent of a communicable severe respiratory tract infection in humans, officially named by the World Health Organization (WHO) as COVID‐19. As of December 19, 2021, 274 million confirmed cases, including 5.36 million deaths, have been reported globally. Due to this global health crisis, enormous financial resources, manpower, and materials have been invested in preventing the further spread of the virus and developing effective strategies for the detection, prevention, and treatment of COVID‐19.

However, since late 2020, the emergence of sets of SARS‐CoV‐2 mutations with modified infectivity, transmissibility, and antigenicity largely impacted the effectiveness of vaccines and therapeutic drugs. The Global Initiative on Sharing All Influenza Data (GISAID) provides a platform for virus genomic sequences, where more than one million SARS‐CoV‐2 sequences are available. Thereby, the biological characteristics of SARS‐CoV‐2 and strategies against this virus and its mutations should be updated in time to help control viral spread.

**FIGURE 1:**
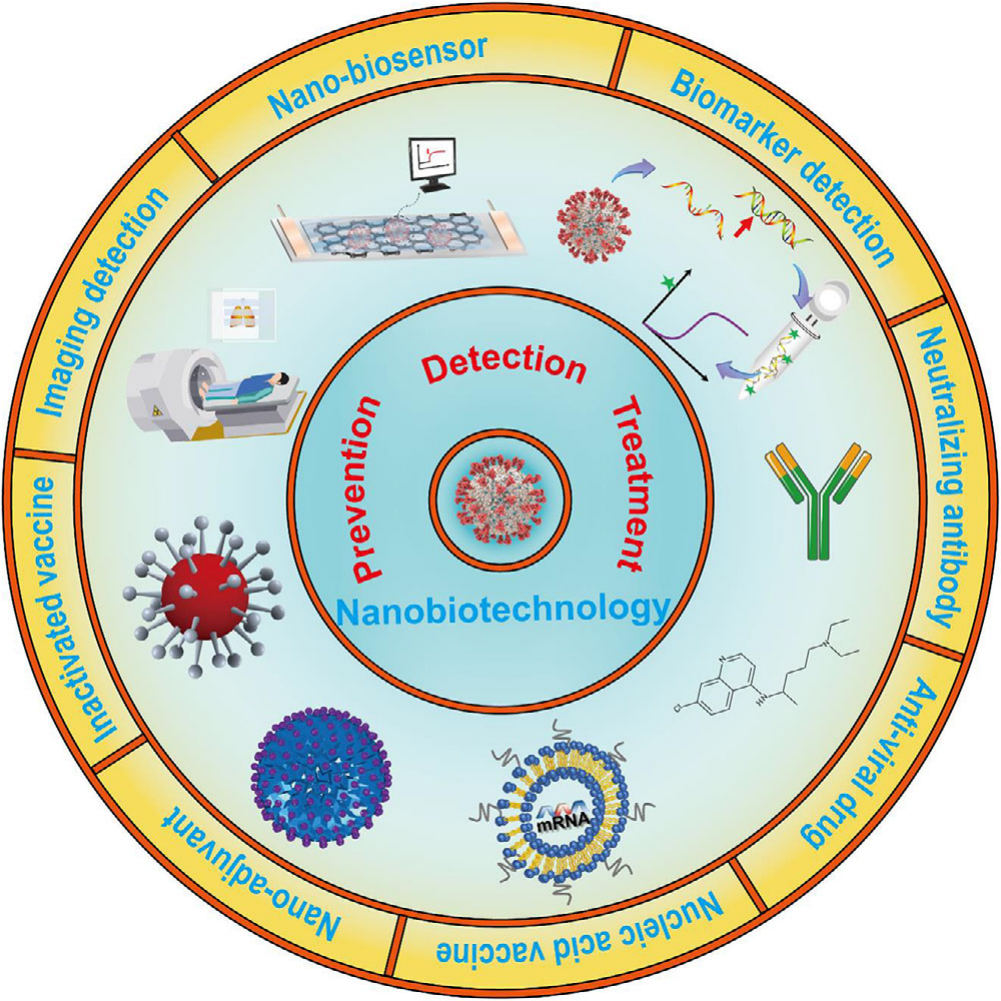
Outline of this review. Strategies that have been explored against COVID‐19 are thoroughly reviewed: (1) detection of COVID‐19 by reverse transcription polymerase chain reaction (RT‐PCR), computed tomographic (CT) imaging, and nanobiosensors (e.g., graphene‐based sensors); (2) prevention of COVID‐19 by inactivated virus vaccine, nucleic acid vaccine, and nanoparticles (e.g., particulate alum); (3) treatment of COVID‐19 by antiviral agents, neutralizing antibodies, and nanotherapeutics

In view of the increasingly severe public health threat posed by COVID‐19, this review aims to give a general overview of this disease and its causative agent, that is, SARS‐CoV‐2. This review focuses on the strategies for detecting, preventing, and treating COVID‐19 and illuminates the underlying mechanisms (Figure 1). Moreover, state‐of‐the‐art studies of nanotechnology‐based promising strategies against COVID‐19 are contained, which will advance the development in managing COVID‐19 and other pandemics in the future. Although some reviews on COVID‐19 have been published, this review is unique in many ways as it systemically and comprehensively summarizes the whole spectrum of the strategies for fighting COVID‐19 and emphasizes the opportunities for nanotechnologies.

## 2 VIROLOGY OF SARS‐CoV‐2

**FIGURE 2:**
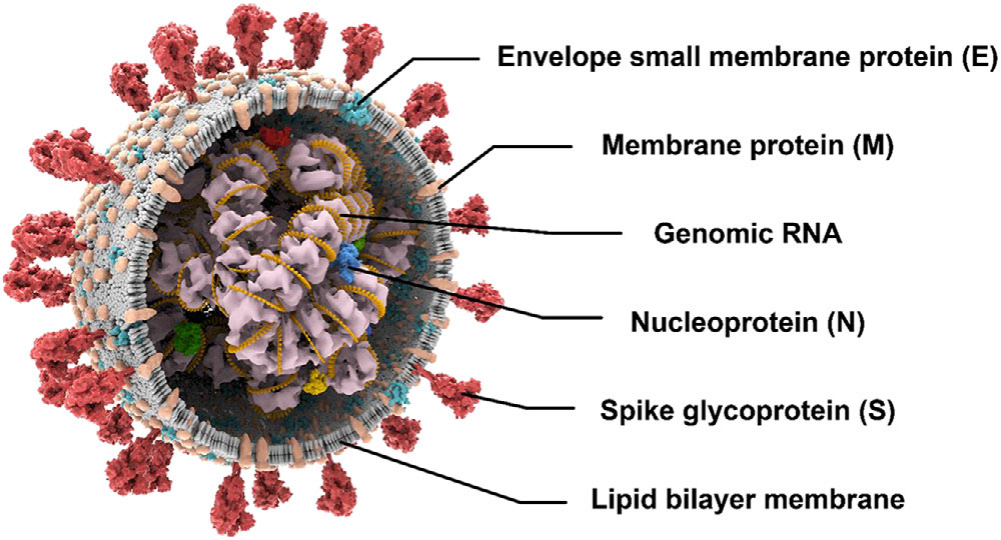
Schematic of SARS‐CoV‐2 structure

SARS‐CoV‐2 is a member of a large family named coronaviridae which includes four genera– alphacoronavirus (α‐CoV), betacoronavirus (β‐CoV), gammacoronavirus (γ‐CoV), and deltacoronavirus (δ‐CoV). SARS‐CoV‐2 is a betacoronavirus in the same genus as SARS‐CoV and Middle East Respiratory Syndrome Coronavirus (MERS‐CoV).^1^ Whole genome sequencing of SARS‐CoV‐2 showed a similarity of 51.8% and 79.0% to MERS‐CoV and SARS‐CoV, respectively.^2^, ^3^ The genetic material of SARS‐CoV‐2 is a single‐stranded positive sense RNA with the size ranging from 26 to 32 kilobases (Figure 2). The genome encodes 24–27 viral proteins, such as four structural proteins and nonstructural proteins.

The four structural proteins of this novel virus include the small envelope protein (E), the nucleocapsid protein (N), the matrix protein (M), and the spike protein (S).^4^ The E protein plays an essential role in the processes of assembly and budding. The N protein could bind to the RNA of SARS‐CoV‐2 and support the formation of the nucleocapsid. The M protein is an integral glycoprotein maintaining the integrity of SARS‐CoV‐2 with other structural proteins. The S protein is a type I trimeric transmembrane protein that determines the host tropism of coronaviruses through binding to the receptors of host cells. The ectodomain of spike protein is cleaved by proteases into two subunits: S1 subunit and S2 subunit. S1 subunit is highly variable between genera with the receptor‐binding activities. At the same time, the S2 subunit is more conserved with the ability to catalyze membrane fusion. On mature viruses, the spike protein exhibits three S1 heads sitting on top of an S2 stalk.^5^ The S1 subunit consists of two domains: an N‐terminal domain (NTD) and a receptor‐binding domain (RBD). The NTD shows a structural fold as human galectins, and the RBD is further divided into two subdomains: internal and external subdomains. The internal sub‐domain is a five‐stranded antiparallel β‐sheet, and the external subdomain contains an actual receptor‐binding motif (RBM) responsible for the receptor‐binding specificity. In the case of SARS‐CoV‐2 and SARS‐CoV, the external subdomain of RBD determines the direct viral binding to the host receptor angiotensin‐converting enzyme 2 (ACE2).^6^, ^7^ The S2 subunit is divided into a transmembrane domain (TM), cytoplasmic domain (CP), conserved fusion peptide (FP), and heptad repeat (HR) 1 and 2.^8^

**FIGURE 3:**
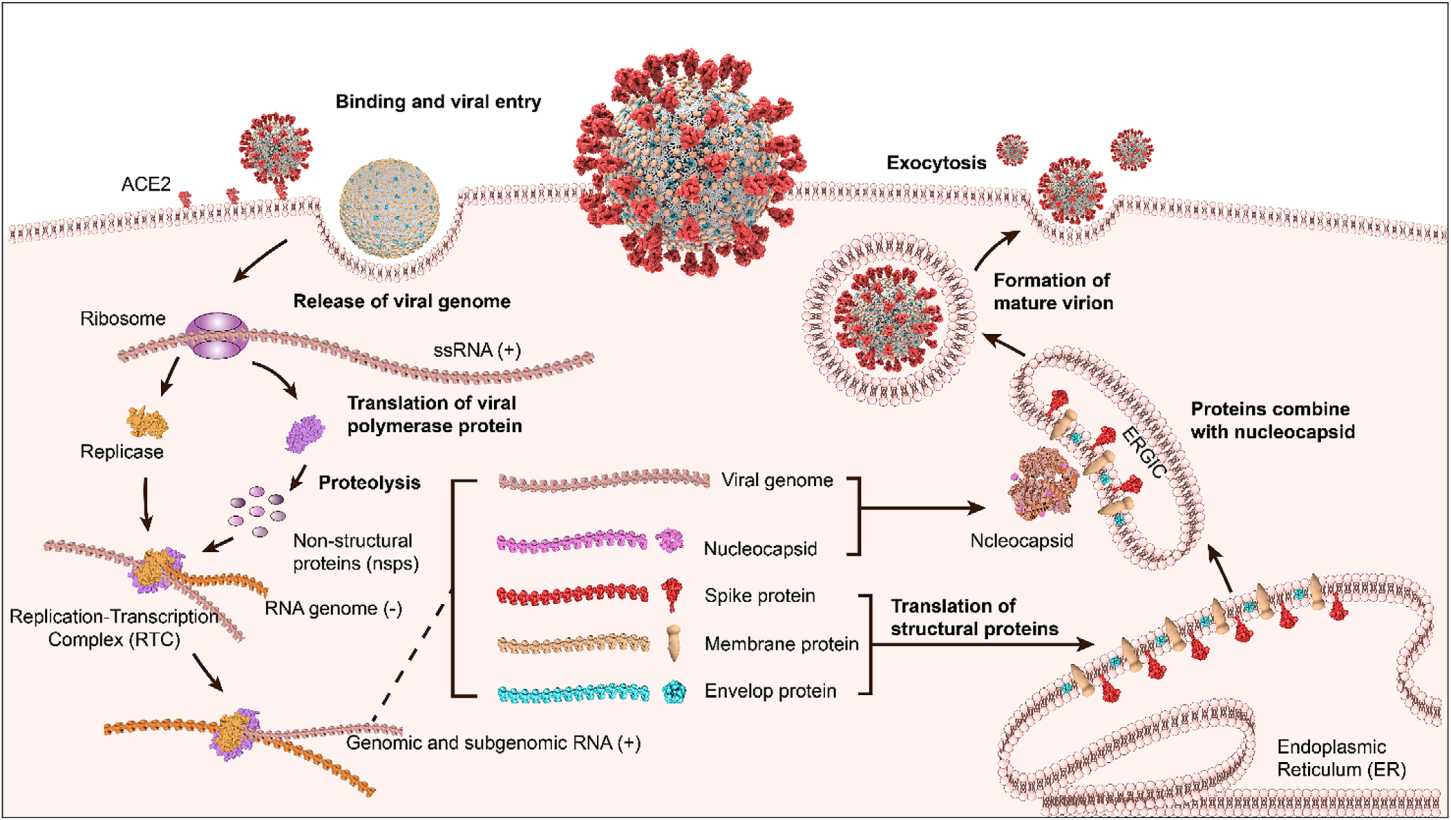
Schematic of SARS‐CoV‐2 life cycle

The life cycle of SARS‐CoV‐2 begins when it binds to the host cell surface through the interaction of its structural S protein with host receptors. ACE2 is the major host cell receptor of SARS‐CoV‐2. Additionally, some investigations reported that SARS‐CoV‐2 spike protein could also interact with host cell receptors CD147 and neuropilin‐1 to facilitate viral infection.^9^, ^10^ After receptor binding, the S protein is cleaved into S1 and S2 subunits by host cell proteases, such as furin, cathepsin, and TMPRSS2. Then, the viral genome is released into the cytosol through S2‐assisted fusion of cellular and viral membrane. In the cytosol, the viral genome is translated to the replicase, followed by the synthesis of viral RNA and the translation of viral proteins. Viral structural proteins (E, M, and S) enter the endoplasmic reticulum (ER), followed by moving into the endoplasmic reticulum‐Golgi intermediate compartment (ERGIC). Then, multiple copies of the N proteins are combined with genomic RNA forming ribonucleoprotein complexes with helical structure. In ERGIC, ribonucleoprotein complexes interact with viral structural proteins (E, M, and S) to form the complete virus particles. Finally, virus particles are released out of the cells via the constitutive exocytic pathway (Figure 3).

## 3 EPIDEMIOLOGY OF COVID‐19

Since only a fraction of severe infections is reported and counted, the overall burden and prevalence of COVID‐19 have been largely underestimated.^11^ According to the results of large‐scale seroprevalence studies, the estimated number of infections exceeds the number of reported cases by approximately 10‐fold or more.^12^, ^13^ Epidemic investigations among the first confirmed cases identified that about 70% of patients had exposure to Huanan seafood market that sold live animals.^14^ Therefore, this market was initially assumed as the source of zoonotic infections.^15^ However, phyloepidemiologic investigations suggested that COVID‐19 was imported from elsewhere and spread silently from human to human before the outbreak in the Huanan market.^16^

The basic reproduction number ($R_{0}$) is an indication of viral transmissibility. It is defined as the average number of new cases generated directly by an infected individual in a population with no immunity. If the $R_{0}\;$< 1, it is suggested that each infectious person generates less than one new infection. Therefore, an epidemic is likely to die out. However, when the $R_{0}\;$> 1, the epidemic is likely to grow. Along with the development of the epidemic, the $R_{0}$ value can change over time. The $R_{0}$ was estimated to be between 2.0 and 3.0 for SARS and around 0.69 for MERS.^17^, ^18^ For SARS‐CoV‐2, the $R_{0}$ was preliminarily reported as 2.24–3.58.^19^ Due to the different stages of the epidemic and distinct estimation methods, several different $R_{0}$ values of SARS‐CoV‐2 were reported. In February 2020, Liu et al. estimated the average $R_{0}$ to be 3.28 and median to be 2.79 using stochastic and statistical methods.^20^ In clinical studies, Yang et al. surveilled 4021‐cases of COVID‐19 identified before January 26, 2020, and reported an $R_{0}$ of approximately 3.77.^21^

COVID‐19 has been characterized as a type of self‐limiting infectious disease, and this disease can occur in five different patterns. The first pattern is asymptomatically infected people without any symptom or clinical sign. The second pattern is mild to medium cases with mild upper respiratory tract symptoms or pneumonia. The third pattern is severe cases with severe pneumonia requiring oxygen administration. The fourth pattern is critical cases with acute respiratory distress syndrome (ARDS) or other acute symptoms such as shock and respiratory failure. The fifth pattern is death. From initial exposure to an infectious agent to the onset of any symptoms, this interval is called the incubation period. A long incubation period may contribute to a high rate of subclinical and asymptomatic infection. The mean incubation period of COVID‐19 is between 4 and 7 days, and an unusual 19‐days incubation case was also reported.^22^ Considering the 19‐days incubation period is a small probability event, the maximum quarantine time was suggested as 14 days.

The COVID‐19 infected person is the main source of infections. The person‐to‐person transmission can occur in several different ways, including contact and droplet transmission,^5^, ^14^, ^24^, ^25^ airborne transmission,^26^, ^27^, ^28^, ^29^ and fomite transmission.^30^, ^31^, ^32^ Additionally, SARS‐CoV‐2 RNA detected by reverse transcription polymerase chain reaction (RT‐PCR) can also be found in other biological samples, including plasma, serum, urine, and feces. Some studies reported that SARS‐CoV‐2 was cultured from stool specimens.^33^ The existence of viable SARS‐CoV‐2 in the urine sample^34^ and blood cells is also reported. Due to limited data, the role of SARS‐CoV‐2 transmission through these routes remains uncertain.

## 4 DETECTION OF COVID‐19

Early and accurate diagnosing is crucial to control the spread of COVID‐19. The symptoms of COVID‐19 include fever, cough, fatigue, sputum production, and shortness of breath, which are similar to other respiratory infections.^35^ Therefore, syndromic testing is not suitable for accurate detection and screening of COVID‐19. The current diagnostic tests include biomarkers detections and imaging detections.

### 4.1 Biomarkers detections

Detection of COVID‐19 by biomarkers relies on molecular techniques to target and identify specific biomarkers. The biomarkers of SARS‐CoV‐2 mainly involve nucleic acid and viral proteins. Compared with the detection of viral protein, nucleic acid testing has been applied more widely to the accurate diagnosis of COVID‐19 in the clinic.

At the initial outbreak of the COVID‐19 pandemic, the full genome of the causative agent (SARS‐CoV‐2) was identified using the combination of RT‐PCR and metagenomic next generation sequencing (NGS).^36^, ^37^, ^38^ Thereby, infectious cases can be confirmed using genome sequencing. However, this strategy is costly, time‐consuming, and labor‐intensive. Many RT‐PCR test kits have been developed to solve these problems with the increased understanding of viral genomic composition. Since the first report of the full SARS‐CoV‐2 genome published on January 7, 2020, there have been many released SARS‐CoV‐2 sequences under the GISAID.^37^ These data are bedrocks for the development of RT‐PCR or another nucleic acid testing. Up to now, RT‐PCR detection is predominantly used for molecular diagnosis of COVID‐19. The National Medical Products Administration has approved more than 10 nucleic acid testing methods for detecting COVID‐19 in China.

An accurate diagnosis using RT‐PCR needs sufficient preliminary work, including the collection and treatment of specimens, sequence alignment, primer design, and assay optimization. In the diagnosis of COVID‐19, respiratory specimens are usually collected because they have the highest viral loads of SARS‐CoV‐2 and highest positive rates of RT‐PCR testing compared with other types of clinical specimens such as blood, stool, and urine.^39^ Respiratory specimens can be divided into upper respiratory specimens and lower respiratory specimens. The upper respiratory specimens include nasopharyngeal and oropharyngeal swabs, as well as nasopharyngeal and endotracheal washes. The lower respiratory specimens include bronchoalveolar lavage, endotracheal aspirate, and sputum. Nasopharyngeal swabs are generally collected to detect COVID‐19 since this approach is less invasive and the maximum storage time of specimens (5 days) is longer than nasopharyngeal or endotracheal washes (2 days).

In the typical process of RT‐PCR testing, the viral RNA is reversely transcribed into cDNA, followed by exponential amplification of specific regions of cDNA.^40^, ^41^ The quantity of the amplified DNA can be detected using fluorescence probes (e.g., TaqMan probe) or double‐stranded DNA binding dyes (e.g., SYBR Green). When a new strand of DNA is synthesized, the polymerase with 5′ exonuclease activity cleaves a TaqMan probe. Then fluorescence signal is produced as the quencher is removed. Thereby, the fluorescence intensity reflects the quantity of the target DNA. Compared with TaqMan probes, SYBR Green displays lower specificity since it can bind with any sequence of dsDNA and produce fluorescence signals.

Thereby, the choice of targets is vital for the specific detection of SARS‐CoV‐2. Many versions of RT‐PCR kits are designed with different targets, including the N gene, the E gene, the S gene, and the RNA‐dependent RNA polymerase (RdRP) gene located within the open reading frame lab (Orflab).^42^, ^43^ The N and E genes can be designed to detect numerous coronaviruses because both of them are highly conserved.^44^ The RdRP gene and S gene are used to specifically detect SARS‐CoV‐2.^6^ Thereby, the RT‐PCR testing can be performed in a two‐step assay; one primer differentiates coronaviruses from other viruses, whereas a second primer is used to distinguish SARS‐CoV‐2 from coronaviruses.

Although the RT‐PCR testing is the most largely used strategy for the molecular diagnosis of COVID‐19, this method cannot be used in point‐of‐care (POC) applications because of its requirements of controlled temperature cycling. To solve this limitation, a large number of alternative exponential amplification techniques that can be accomplished at a single temperature have been developed. These techniques include recombinase polymerase amplification (RAP), exponential amplification reaction (EXPAR), rolling circle amplification (RCA), loop‐mediated isothermal amplification (LAMP), and exponential strand displacement amplification (E‐SDA).

### 4.2 Imaging detections

Computed tomographic (CT) of the chest can be used temporarily for the detection of COVID‐19 in the clinic. This method is to solve the problem of shortage and the high false‐negative rate of RT‐PCR kits. CT scans are fast, accurate, painless, and non‐invasive.^45^, ^46^ This strategy involves using special X‐ray equipment to measure a patient's chest at different angles. Thereby, cross‐sectional images of the chest with high resolution can be obtained through chest CT. Radiologists examine these images to find abnormalities and to diagnose the diseases. The typical features of COVID‐19 from CT imaging are distinct at different stages of the disease. On most prominent 0–4 days after the onset of symptom, bilateral, and peripheral ground‐glass opacities in the lung are the hallmark features of COVID‐19, followed by rounded opacities, enlarged intra‐infiltrate vessels, and later increasing consolidations. The consolidations of the lungs refer to the fluid or solid material in lung tissue, which is a symptom of progressing critical illness.^47^, ^48^ Depended on these imaging features, CT scans have shown higher sensitivity than RT‐PCR. However, a low specificity has been demonstrated as a limitation of chest CT, because the imaging feature of COVID‐19 overlaps with other influenza‐associated pneumonia.

**FIGURE 4:**
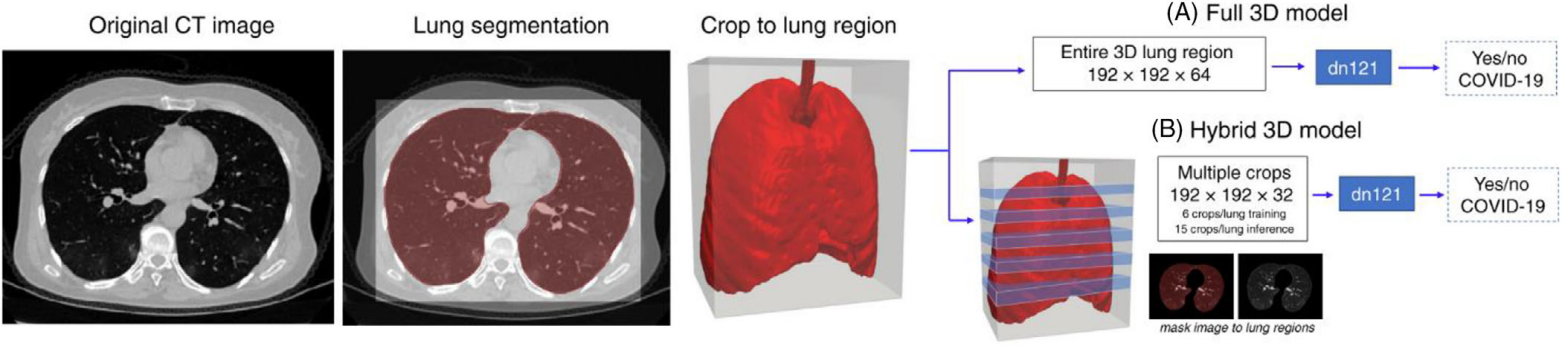
Schematic of three‐dimensional (3D) classification workflow. Reproduced with permission.^49^ Open access

Artificial intelligence (AI) approaches have been explored to detect and characterize COVID‐19 on imaging to overcome this obstacle. For example, Harmon et al. developed a series of deep learning algorithms trained in 1280 patients from a different country to classify COVID‐19 (Figure 4).^49^ After training, 1377 patients were utilized for algorithms evaluation by two classification models. One was named full three‐dimensional (3D) model using the whole lung region with fixed input size, and another was termed hybrid 3D model using the average score of multiple regions of the lung at a fixed image resolution. The accuracy, sensitivity, and specificity of model classification achieved up to 90.8%, 84%, and 93%, respectively.

## 5 PREVENTION OF COVID‐19

As an effective way to protect susceptible persons and block the spread of infectious diseases, vaccines have always been the primary weapon for humans to fight infectious diseases. Since there are no effective drugs now for the COVID‐19 therapy, the development of the vaccine is particularly important. Researchers all over the world are trying different approaches to developing a vaccine. In what follows, we discuss various methods based on the fabrication techniques or vaccine platforms currently used worldwide for creating vaccine candidates.

### 5.1 Virus vaccines

As the most common approach to fabricating vaccines, virus vaccines have been traditionally used to prevent infectious diseases. In this approach, the live virus should be isolated first. Then, careful processing will be followed to make the virus pathogenicity disappear. After the strict test of safety verification, the virus itself will be used directly to induce potent immune responses. According to the processing method, virus vaccines consist of inactivated vaccines and live‐attenuated vaccines.

**FIGURE 5:**
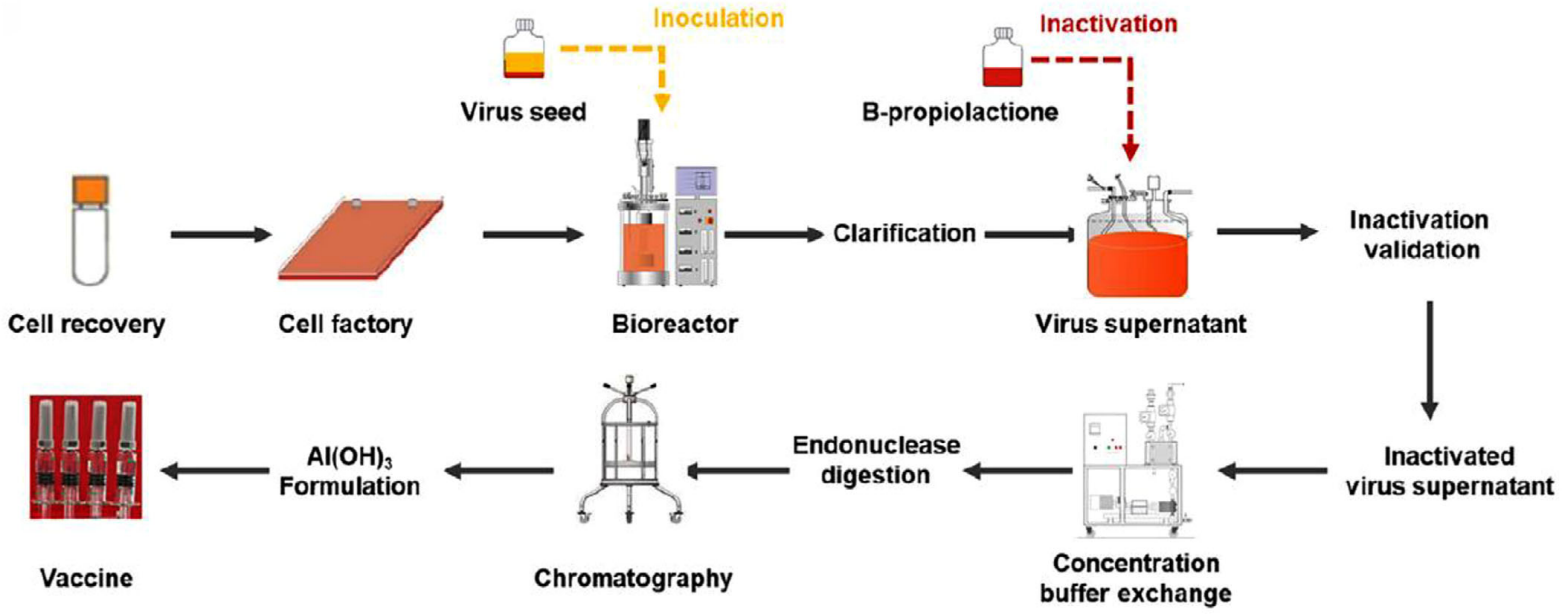
Process flow of an inactivated vaccine, BBIBP‐CorV. Reproduced with permission.^50^ Copyright 2020, Elsevier

Compared with live‐attenuated vaccines, the viruses used for inactivated vaccines lose their infectivity completely. A relatively faster development speed also makes inactivated vaccines a powerful prevention strategy for COVID‐19. An inactivated vaccine, BBIBP‐CorV, has been approved by WHO to be included in the global emergency use authorization (EUA) list. It used a SARS‐CoV‐2 strain HB02, isolated from a hospital patient, to be the virus seed (Figure 5).^50^ The strain was cultured and passaged via infecting Vero cells in vitro and expanded to generate the virus stock in a basket bioreactor. Its genetic stability was monitored carefully during the virus stock production (Figure 5). The stock viruses were inactivated by β‐propionolactone to eliminate their infectivity. Then, the inactivated virus supernatant was concentrated and purified. The final vaccine was formed by mixing inactivated viruses with aluminum hydroxide adjuvant (Figure 5). Preclinical tests demonstrated that BBIBP‐CorV could induce high levels of neutralizing antibodies in different animal models.^1^ The phase 1/2 and phase 3 trials indicated good safety and an effective vaccine for COVID‐19 of this inactivated SARS‐CoV‐2 vaccine.^51^, ^52^ The main challenge for inactivated vaccines is an antibody‐dependent enhancement (ADE)^53^ of infection. It has a theoretical probability for any therapy or therapy based on an antibody to enhance the viral infection. For the BBIBP‐CorV and another leading inactivated vaccine, CoronaVac, ,^54^ the ADE had not been observed both in preclinal^1^ and clinical trials.^51^, ^52^, ^54^

### 5.2 Nucleic acid vaccines

Nucleic acid‐based therapy for the prevention of infectious diseases has made significant progress in recent years. Nucleic acid vaccines, including DNA and RNA, are attractive approaches for preventing COVID‐19 due to the rapid development cycle from design to clinical trial. In fact, mRNA‐1273, a kind of messenger RNA (mRNA) vaccine against SARS‐CoV‐2 developed by Moderna, initiated the phase I trial just 66 days after the viral sequence was released.^55^ Nucleic acid vaccines are easy to manufacture; only genomic sequences of SARS‐CoV‐2 are needed before the vaccine design and production. The DNA or RNA encoding the viral antigen gene fragment is directly used to develop vaccines. After they enter human cells, the viral antigen protein will be expressed and induce the immune responses.^55^, ^56^ The big challenge for nucleic acid vaccines is that the therapeutic effects have yet to be proven, as no vaccines are licensed using this approach before the global pandemic of COVID‐19.^57^

To trigger an immune response, virus vaccines as inactivated or live‐attenuated pathogenic microorganisms are injected into our bodies. However, the principle of using mRNA as COVID‐19 vaccines is somewhat different. The synthetic mRNA is used to instruct body cells how to produce a protein or a protein fragment derived from SARS‐CoV‐2 that triggers an immune response in vivo.^58^, ^59^ Then, the immune system recognizes that the protein does not belong to our own body, which triggers our immune system to produce specific antibodies and activate other immune cells to fight this pseudo‐infection. When the authentic SARS‐CoV‐2 enters our body, the immune system protects us from being infected.^55^, ^56^ RNA vaccines do not have to enter the nucleus for transcription compared with DNA vaccines. They can directly synthesize proteins in the cytoplasm and reduce the risk of viral DNA integration into host DNA. BNT162b1 is a kind of mRNA vaccine which encodes the RBD of the spike glycoprotein of SARS‐CoV‐2.^56^ To increase the immunogenicity of the vaccine, a foldon trimerization domain derived from T4 fibritin is added to the antigen domain. The genetic materials are packaged into lipid nanoparticles to form the vaccines.^56^ The lipid coat can help the mRNA enter cells efficiently. Phase I/II study confirmed strong antibody responses and robust immune T cell responses.^60^ It is the advantage of the nucleic acid vaccine that it can induce not only humoral immunity but also cellular immunity. Further phase III trial using the BNT162b2 vaccine, which encodes a full‐length spike glycoprotein of SARS‐CoV‐2 virus, indicates 95% efficiency for preventing COVID‐19.^61^ Additionally, the insufficient mRNA stability also makes the storage and transportation of RNA‐based vaccines very troublesome. A piece of cold‐chain equipment for ultra‐low temperature up to −20°C or −70°C is needed. More thermostable mRNA vaccines are conducive to the promotion of vaccines to prevent COVID‐19. The efficiency of ARCoV, another RBD‐encoding mRNA vaccine, has been evaluated in the preclinical studies after the vaccine was stored at 4°C or 25°C for 7 days. The long‐term stability and clinical trial are on the way.^62^

### 5.3 Viral‐vector vaccines

Viral‐vector vaccines are developed on some replication‐deficient viruses, such as non‐replicating adenovirus or weakened replicating measles. The genome of these viruses is genetically engineered to load immunogenic proteins of SARS‐CoV‐2 into their framework. After vaccination, they can produce coronavirus proteins in the human cells. It is similar to the mRNA vaccine for viral‐vector vaccines to work in the body. But the medium or vector of immunogenic proteins is another virus. In theory, viral‐vector vaccines can also induce multiple immune responses. Researchers have developed a ChAdOx1‐based adenoviral vector vaccine after the outburst of COVID‐19.^63^, ^64^ The whole length of spike proteins of the SARS‐CoV‐2 can be encoded by this vaccine. A balanced humoral and cellular immune response was observed in rhesus macaques after vaccination.^63^ Clinical trials indicated sufficient immunogenicity and acceptable safety of the vaccine.^64^, ^65^ Another vaccine developed by CanSinoBio, Ad5‐nCoV, uses the non‐replicating adenovirus as a vector.^66^, ^67^ Phase I clinical trials showed the vaccine's good toleration and immunogenicity. Further randomized controlled phase II trial confirmed effective immune responses after a single vaccination and no serious adverse effects.^68^ The main challenge for viral‐vector vaccines is the preexisting antibody effect. The population may have a relatively high infection rate for the virus used for vaccine vectors, such as the adenovirus.^66^ The preexisting antibody can slow down the immune response induced by the vaccine.

### 5.4 Protein‐based vaccines

According to the summary information of WHO, the number of protein‐based vaccine candidates is the most in clinical development by June 25, 2021.^69^ Most of these vaccines are protein subunits, and the others are virus‐like particles (VLP). The key technology or challenge of these vaccines is the production of recombinant proteins or VLPs with high immunogenicity. Due to the predominant role of entering cells, most of the protein‐based vaccines for COVID‐19 focus on the spike protein or the RBD domain of SARS‐CoV‐2. One of the leading protein subunits‐based vaccine candidates, VX‐CoV2373, was developed based on the full‐length spike protein.^70^, ^71^ After expression and purification from insect cells, the protein was mixed with polysorbate 80 detergent to form the vaccine. The efficacy and safety of this vaccine have been evaluated by phase I–II clinical trial. The higher efficacy of protein‐based vaccines was demonstrated among HIV‐negative participants,^70^ indicating the promise of using this type of vaccines for preventing COVID‐19.

## 6 TREATMENT OF COVID‐19

### 6.1 Antiviral agents

The development of a new treatment of novel diseases is usually time‐consuming. Up to date, no specific treatment or drug for the COVID‐19 infection has been identified. Therefore, the predeveloped antiviral drugs with broad‐spectrum effects could be utilized as an effective strategy to meet an emergency. These drugs inhibit coronavirus replication by targeting the critical proteins in the pathogenesis and life cycle of the virus, such as the spike protein of SARS‐CoV‐2, RdRP, papain‐like protease, and 3‐chymotrypsin‐like protease.

Griffithsin and nafamostat could block the replication of SARS‐CoV‐2 through inhibiting the virus attachment and cell entry. Griffithsin, a broad‐spectrum antiviral drug, could bind to various glycoproteins on the viral surface, such as the glycoprotein 120 of HIV and the spike glycoprotein of SARS‐CoV.^72^, ^73^, ^74^ Due to the high similarity across the spike protein of SARS‐CoV and SARS‐CoV‐2,^75^ griffithsin constitutes a potential treatment choice for COVID‐19 infections. Nafamostat has also shown an antiviral activity through targeting the host protease TMPRSS2 and inhibiting the virus−host cell membrane fusion.^76^ As mentioned above, TMPRSS2, a cell surface protease, is able to cleave S2 subunits of SARS‐CoV‐2 spike proteins. This process is essential for the membrane fusion of SARS‐CoV‐2 with the host cell. Thereby, nafamostat could be considered as a candidate agent against the SARS‐CoV‐2.

Many antiviral drugs could block the intracellular life cycle of SARS‐CoV‐2 by targeting proteases of the virus to inhibit the viral protein precursors’ cleavage. Disulfiram could specifically bind to the Zn‐bound cysteines in viral proteases including PLpro, NSP10, and NSP13.^77^ Kaletra, the combination of lopinavir and ritonavir, has demonstrated a high binding affinity with 3CLpro proteases of SARS‐CoV‐2 through a computational method.^78^ Additionally, nelfinavir could also bind with the 3CLpro of SARS‐CoV‐2 in a molecular docking study.^79^ Since these proteases are crucial in SARS‐CoV‐2 replications, disulfiram, kaletra, and nelfinavir can be considered as a potential therapeutic choice for COVID‐19.

RdRp is an essential target for the therapeutic of COVID‐19 for its highly conserved structure and crucial function in the replication of viruses.^80^ Many existing drugs could selectively bind to the viral RdRp and inhibit viral RNA synthesis without blocking mammalian host cells' RNA or DNA synthesis. These drugs are almost nucleotide and nucleoside analogs, such as favipiravir,^81^ ribavirin,^82^ remdesivir,^83^ and galidesivir.^84^ Favipiravir and ribavirin are guanosine analogs, while remdesivir and galidesivir are adenosine analogs. In a recent in vitro study, the effects of seven different drugs against SARS‐CoV‐2 were investigated. These drugs include two broad‐spectrum antiviral drugs (remdesivir and favipiravir, targeting RdRp) and five US FDA‐approved drugs. The study verified the effectiveness of remdesivir in the inhibition of the replication of SARS‐CoV‐2.^85^

### 6.2 Chloroquine and hydroxychloroquine

**FIGURE 6:**
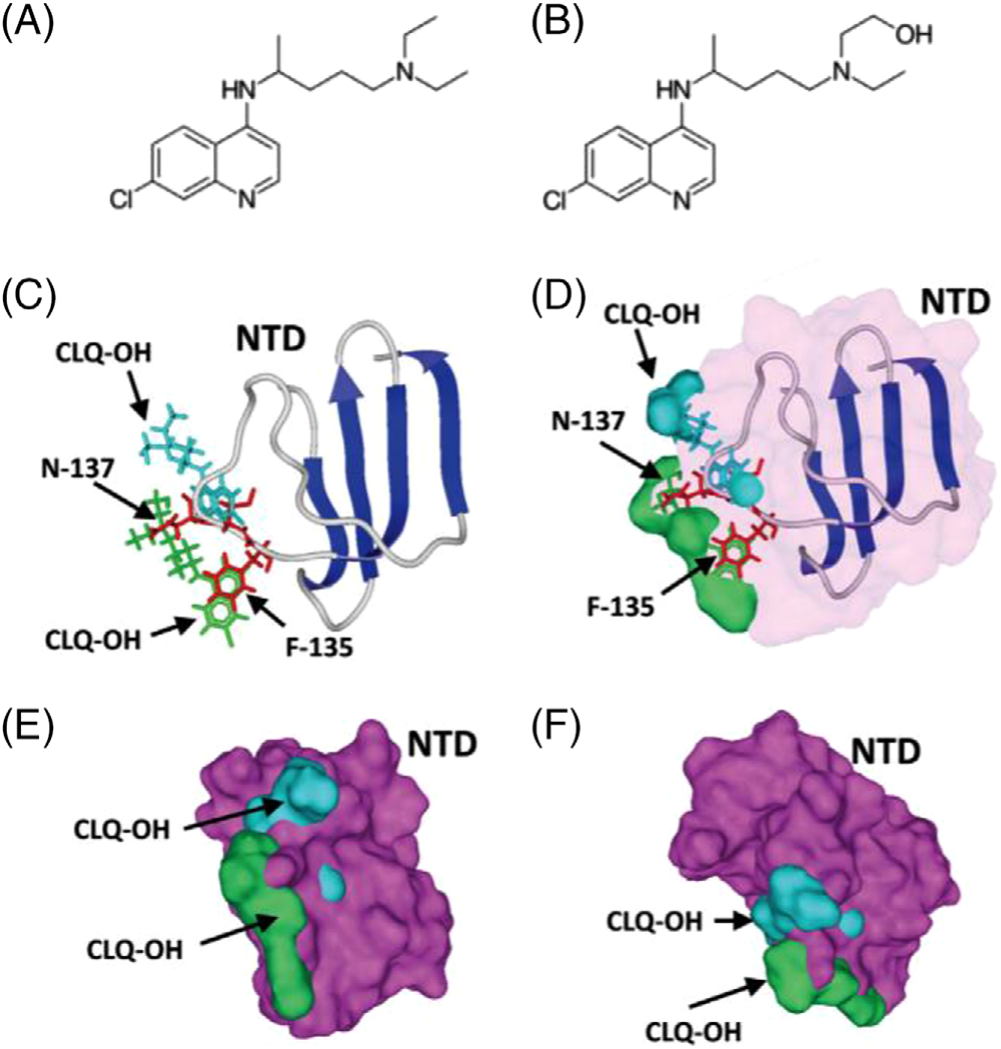
Chemical structure and mechanism of action. (A) Chemical structure of chloroquine. (B) Chemical structure of hydroxychloroquine. (C–F) Two hydroxychloroquine molecules bound to ganglioside GM1 were superposed onto the N‐terminal domain (NTD) bound to GM1. (C,D) The interaction between GM1 and the residues (N‐137 and F‐135, in red) of NTD is blocked by two hydroxychloroquine (in green and blue). (E,F) Superimposition of two hydroxychloroquine molecules (in green and blue) with the NTD surface (in magenta) bound to the ganglioside GM1. Reproduced with permission.^86^ Copyright 2020, Elsevier

Chloroquine and hydroxychloroquine are well‐known antimalarial and autoimmune disease drugs with broad‐spectrum antiviral activity. As shown in Figure 6, the only difference in the chemical structure between chloroquine and hydroxychloroquine is that hydroxychloroquine has one more hydroxyl group at the end of the side chain. Both of them can reduce the viral load of SARS‐CoV‐2. Multiple mechanisms are underlying their antiviral activity. Previous studies reported that both of them could block virus/cell fusion by elevation of endosomal pH. Additionally, chloroquine has been found to interfere with the glycosylation of ACE2, the main cellular receptor of the SARS‐CoV‐2. From a recent publication, chloroquine/hydroxychloroquine has exhibited a high binding affinity to gangliosides and sialic acids. Due to the fact that gangliosides and sialic‐acid‐containing glycoproteins are important attachment factors of the viral replication cycle, chloroquine/hydroxychloroquine are potential blockers of the interaction between the ganglioside on the cellular surface and the spike glycoprotein of SARS‐CoV‐2.^86^

### 6.3 Corticosteroids

Some recent publications showed that corticosteroids could suppress lung inflammation and reduce 28‐day mortality among COVID‐19 patients.^14^, ^87^ However, some harmful clinical outcomes of corticosteroids have also been reported.^88^, ^89^ For the management of MERS and SARS, the patients given corticosteroids were associated with delayed virus clearance. In some cases, corticosteroid treatment causes worse clinical side effects, including psychosis, diabetes, avascular necrosis, and secondary bacterial infection.^90^, ^91^, ^92^ Living guidance from the WHO suggests that only severe and critical patients with COVID‐19 are recommended to use systemic corticosteroids treatment rather than no systemic corticosteroids. In contrast, non‐severe COVID‐19 patients should not use corticosteroids therapy.^93^

### 6.4 Antibodies

**FIGURE 7:**
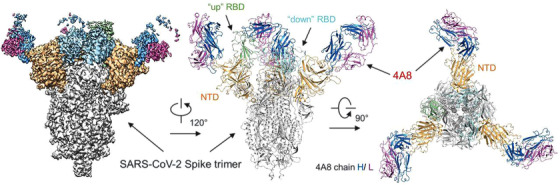
Cryogenic electron microscopy (Cryo‐EM) characterization of the 4A8 formed complex with the spike protein of SARS‐CoV‐2. (A) Molecular surface representation of the complex. (B,C) The overall structure is viewed along two orthogonal axes. The light (in magenta) and heavy (in blue) chains of 4A8 bound to the NTDs (in orange) of spike protein (in grey). The two “down” receptor‐binding domains (RBDs) and one “up” RBD of spike protein are colored cyan and green, respectively. Reproduced with permission.^101^ Open access

Developing therapeutic antibodies with potent neutralizing ability against SARS‐CoV‐2 has important implications. The major inducer of the neutralizing antibody is the spike protein, and the RBD of the spike protein is relatively abundant.^94^ Many monoclonal antibodies (mAbs) targeting the RBD have been reported.^95^, ^96^, ^97^, ^98^, ^99^, ^100^ These mAbs can inhibit the interaction between ACE2 and the spike protein and thus neutralize SARS‐CoV‐2. However, the application of RBD‐binding monoclonal antibodies alone might induce viral resistance mutations. Thereby, cocktail therapeutics for COVID‐19 containing RBD‐targeting antibodies and non‐RBD‐targeting antibodies should be developed. Chen and her colleagues isolated and characterized a mAb, named 4A8, which can target the NTD of the spike protein (Figure 7).^101^ From their results, 4A8 can potently neutralize SARS‐CoV‐2. Thus, it can be used as a therapeutic mAbs against COVID‐19.

### 6.5 Convalescent plasma transfusion

Convalescent plasma transfusion is a therapeutic method, where plasma collected from previously infected individuals is used to treat infected patients. This method has been used for the treatment of respiratory system infections for more than a century.^102^, ^103^, ^104^ The mechanism underlying this treatment is that the potential therapeutic antibodies contained in the convalescent plasma can be transferred to the plasma recipient. A recent publication showed that a lower mortality rate within 30 days after convalescent plasma transfusion is associated with plasma with high antibody titer rather than low antibody titer.^105^ However, the convalescent plasma with a high antibody level is difficult to obtain, limiting the clinical application of this method.

## 7 OPPORTUNITIES FOR NANOBIOTECHNOLOGY TO CONTRIBUTE TO THIS FIELD

The global outbreak of COVID‐19 also requires urgent development of all the available nanotechnology tools. Herein, the status quo of nanotechnology used in the treatment, prevention, and detection of COVID‐19 was systematically presented.

### 7.1 Nanobiotechnology for developing detection techniques

As we have discussed in Section 4, biomarkers and imaging detections are widely used in the diagnosis of COVID‐19. These detection techniques still face restrictions, such as requiring expert human resources and specific instruments. To overcome these limitations, scientists have employed nanomaterials with various unique characteristics to detect COVID‐19.

**FIGURE 8:**
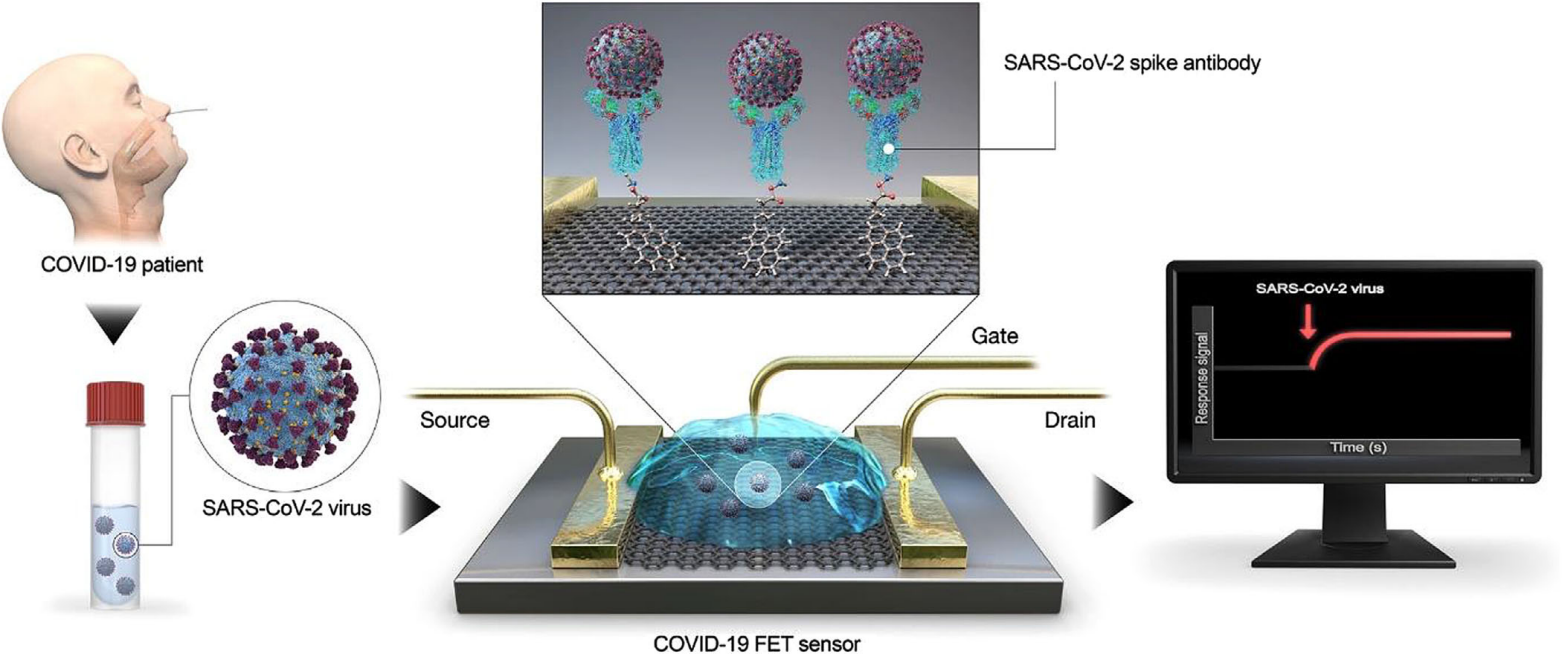
Schematic of graphene‐based field‐effective transistor (FET) biosensor operation procedure. Reproduced with permission.^107^ Open access

Graphene, a novel two‐dimensional material, processes unique characteristics such as large specific area, high carrier mobility, and high electronic conductivity.^106^ Therefore, graphene has been widely used in various sensing platforms. Seo et al. designed a field‐effective transistor (FET) biosensor composed of graphene integrated with the antibody of SARS‐CoV‐2 spike protein for the detection of the virus.^107^ This platform can be applied to detect SARS‐CoV‐2 antigen protein from clinical samples, such as the nasopharyngeal swabs. The limit of detection (LOD) of this platform can achieve 1 fg/ml, suggesting that the nanoplatform can highly sensitively detect SARS‐CoV‐2 in the clinic (Figure 8).

**FIGURE 9:**
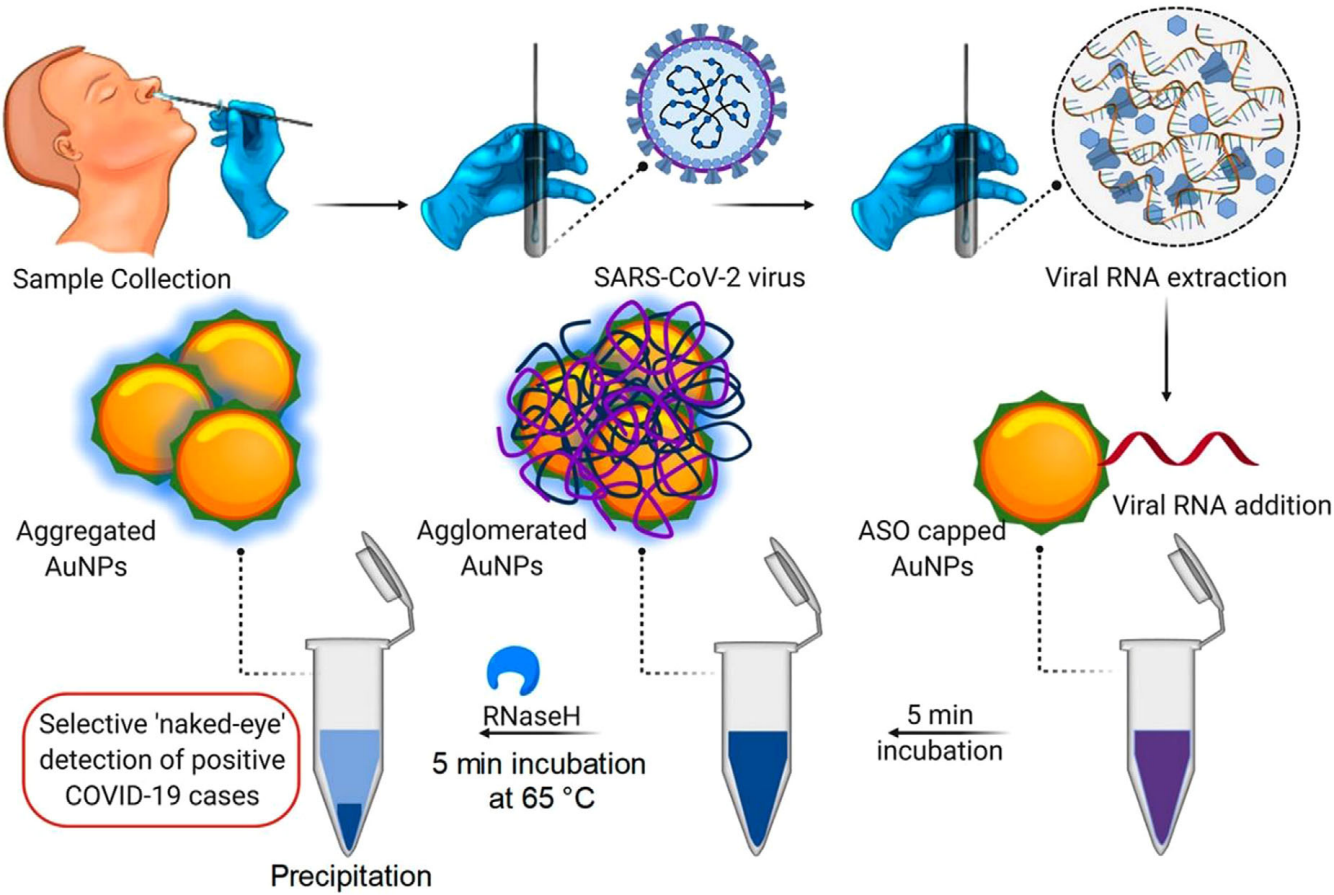
Schematic of gold nanoparticles‐based colorimetric biosensor operation procedure. Reproduced with permission.^108^ Open access

Besides FET biosensors, colorimetric‐based biosensors have also garnered incredible attention in the field of viral detection. Recently, Moitra et al. synthesized a thiol‐modified antisense oligonucleotide (ASOs) capped gold nanoparticles to detect the SARS‐CoV‐2 (Figure 9).^108^ The ASO sequences contain two regions of the nucleocapsid phosphoprotein gene of SARS‐CoV‐2. Therefore, in the presence of nucleic acids of SARS‐CoV‐2, ASOs‐capped gold nanoparticles agglomerated, resulting in a change in its surface plasmon resonance and in color from violet to blue. Furthermore, the addition of RNaseH amplifies the changes, resulting in a “naked‐eye” detectable precipitation of gold nanoparticles. This strategy can be applied to detect COVID‐19 from isolated RNA samples without the requirement of a specific instrument and expert humans.

### 7.2 Nanobiotechnology for developing vaccines

**FIGURE 10:**
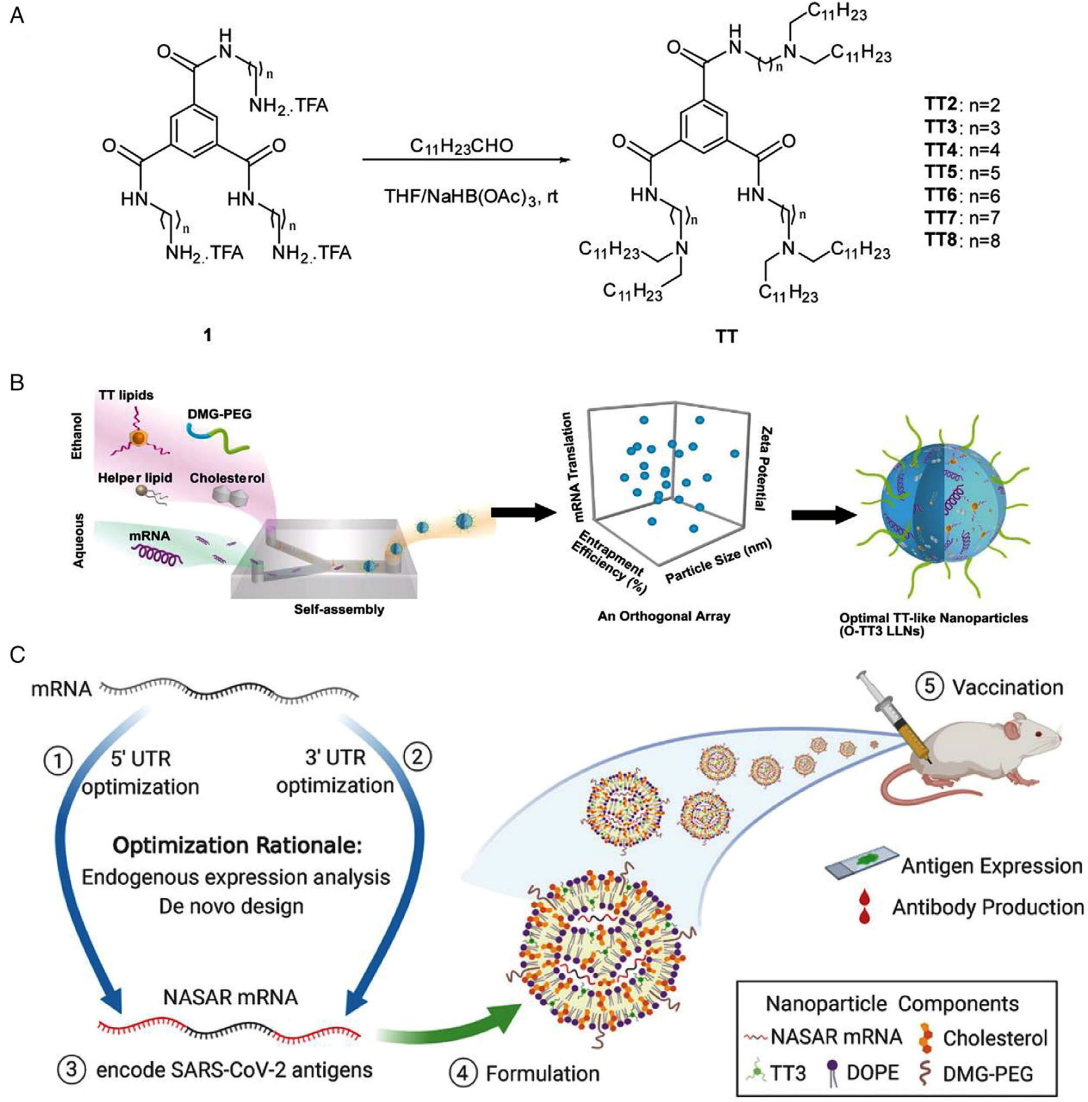
The preparation of TT3 lipid nanoparticles for packing and delivering engineered mRNA as SARS‐CoV‐2 vaccines. (A) The synthetic route to a class of *N* ^1^,*N*,^3^ *N* ^5^‐tris(2‐aminoethyl)benzene‐1,3,5‐tricarboxamide, (TT2–TT8). (B) TT3 lipid nanoparticles were formulated by mixing TT lipids, DMG‐PEG, cholesterol, helper lipids with mRNA to achieve self‐assembly in a microfluidic based device. (A,B) Reproduced with permission.^110^ Copyright 2020, John Wiley and Sons. (C) Schematic of the preparation of engineered mRNA and delivery of the vaccine through TT3 nanoparticles for inducing strong immune reaction against SARS‐CoV‐2 in mice. (C) Reproduced with permission.^111^ Copyright 2015, American Chemical Society

Since the rapid development of nanotechnology, various nanoparticles have been employed to deliver or develop vaccines. For example, lipid nanoparticles play an important role in developing mRNA vaccines, protecting mRNA from degradation, and transporting mRNA to the right localization in vivo.^109^ A lipid nanoparticle, TT3, was developed by Li et al. via an orthogonal array design.^110^ This TT3 nanoparticle contains DMG‐PEG_2000,_ cholesterol, *N* ^1^,*N*,^3^ *N* ^5^‐tris(2‐aminoethyl)benzene‐1,3,5‐tricarboxamide (TT), helper lipids, and mRNA, significantly improving the mRNA vaccine delivery efficiency (Figure 10A,B). From a recently published report, Zeng et al. use TT3 nanoparticles to deliver the optimal untranslated regions of mRNAs for expressing potential SARS‐CoV‐2 antigens.^111^ The TT3‐nanoparticles with mRNAs can induce antigen‐specific antibodies with high efficiency in vaccinated mice (Figure 10C).

**FIGURE 11:**
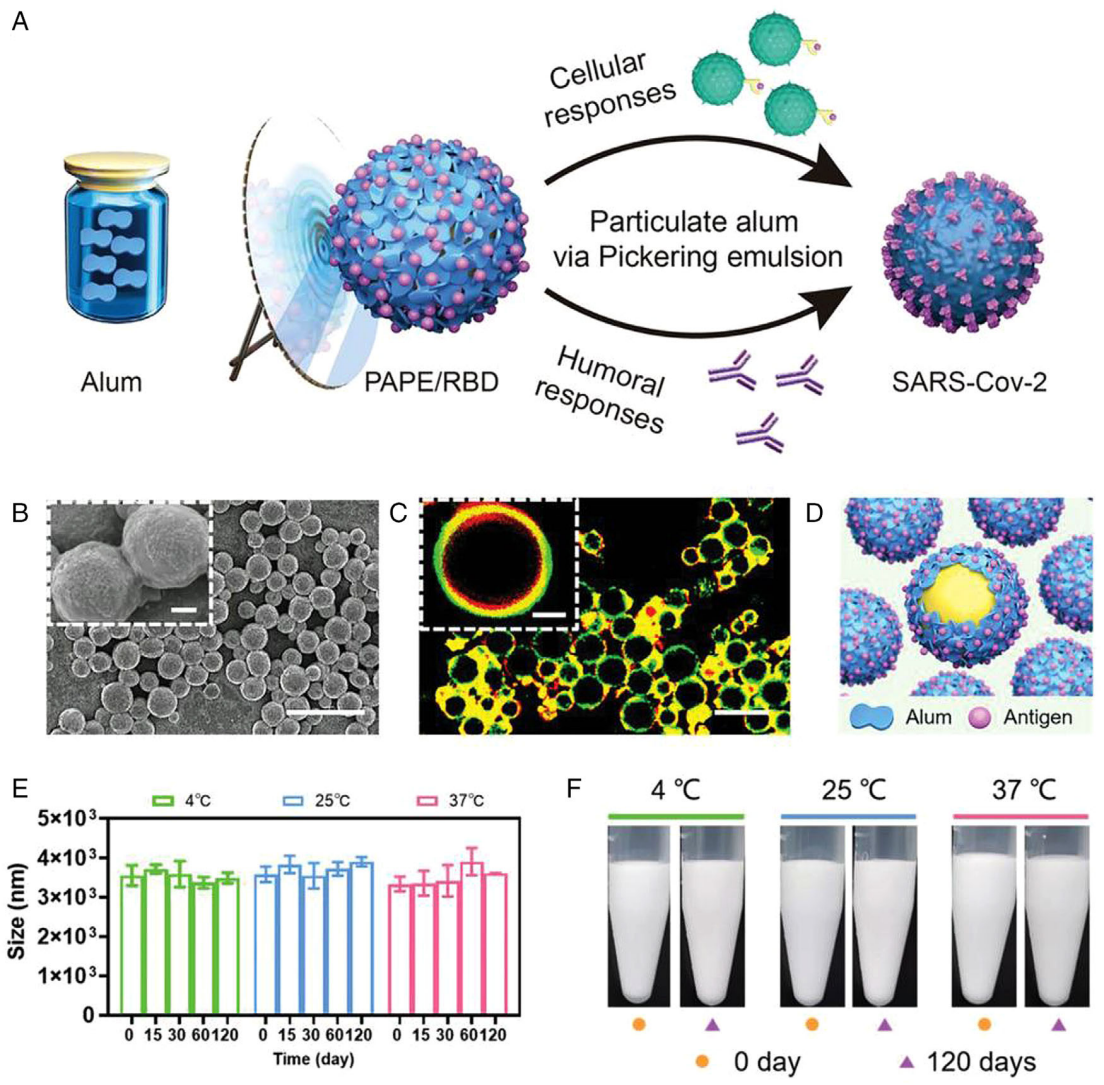
(A) Schematic of the preparation of particulate alum via Pickering emulsion for the application as vaccine adjuvant against SARS‐CoV‐2. (B) The scanning electron microscopy (SEM) image of particulate alum. Scale bars of image and inset represent 10  μm and 200 nm, respectively. (C) The laser confocal image of particulate alum (in red) modified with antigen (in green). (D) Schematic of antigen‐modified particulate alum. (E) Size and (F) schematic images of particulate alum before and after 120 days storage. Reproduced with permission.^112^ Copyright 2020, John Wiley and Sons

Nanotechnology can also be used to deliver immune adjuvants for improving immune activations. For example, Peng et al. performed a study on particulate aluminum hydroxide (alum) for an enhanced vaccine adjuvant against SARS‐CoV‐2.^112^ In their work, a particulate alum (PAPE) was constructed by microgel‐stabilized squalene‐in‐water Pickering emulsion. This particle can largely absorb SARS‐CoV‐2 antigens and enter dendritic cells, triggering the potent humoral and cellular immune reactivities (Figure 11). The in vivo COVID‐19 vaccinations results showed that PAPE‐primed mice generated antibodies and IFN‐α‐secreting T cells six‐fold and three‐fold more than the alum group, respectively.

### 7.3 Nanobiotechnology for developing nanotherapeutics

Based on the rapid development of nanotechnology, many nanomaterials have been explored to overcome the limitations of current antiviral therapy. First, engineered nanomaterials can accumulate and target‐specific pathophysiology sites of COVID‐19 at the organ, cellular, and intercellular level. Second, nanomaterials have large specific surface area which can entrap high payloads of drugs efficiently and protect them from premature release and degradation in a relatively complex physiological microenvironment. Furthermore, the reasonably functionalized nanomaterials provide the controllable release of the loaded drug, mitigating the potential side effects.

Many nanomaterials have shown an intrinsic antiviral activity that can be used to combat viral infection. For example, a nanoplatform was developed using a mixture of titanium dioxide nanoparticles, silver colloid, and a dispersion stabilizer to inhibit the growth of bacteria, fungal, and viruses.^113^ This platform can effectively inhibit the growth of coronaviruses, such as porcine epidemic diarrhea virus (PEDV) and transmissible gastroenteritis virus (TGEV), with a 1000‐fold diluted concentration. Additionally, the Chen group has investigated the antiviral activity of four silver nanomaterials against TGEV.^114^ From their results, silver nanoparticles and two silver nanowires with different lengths are capable of protecting swine testicle cells against TGEV infection, while silver colloids exhibited no inhibitory effect on TGEV infection.

**FIGURE 12:**
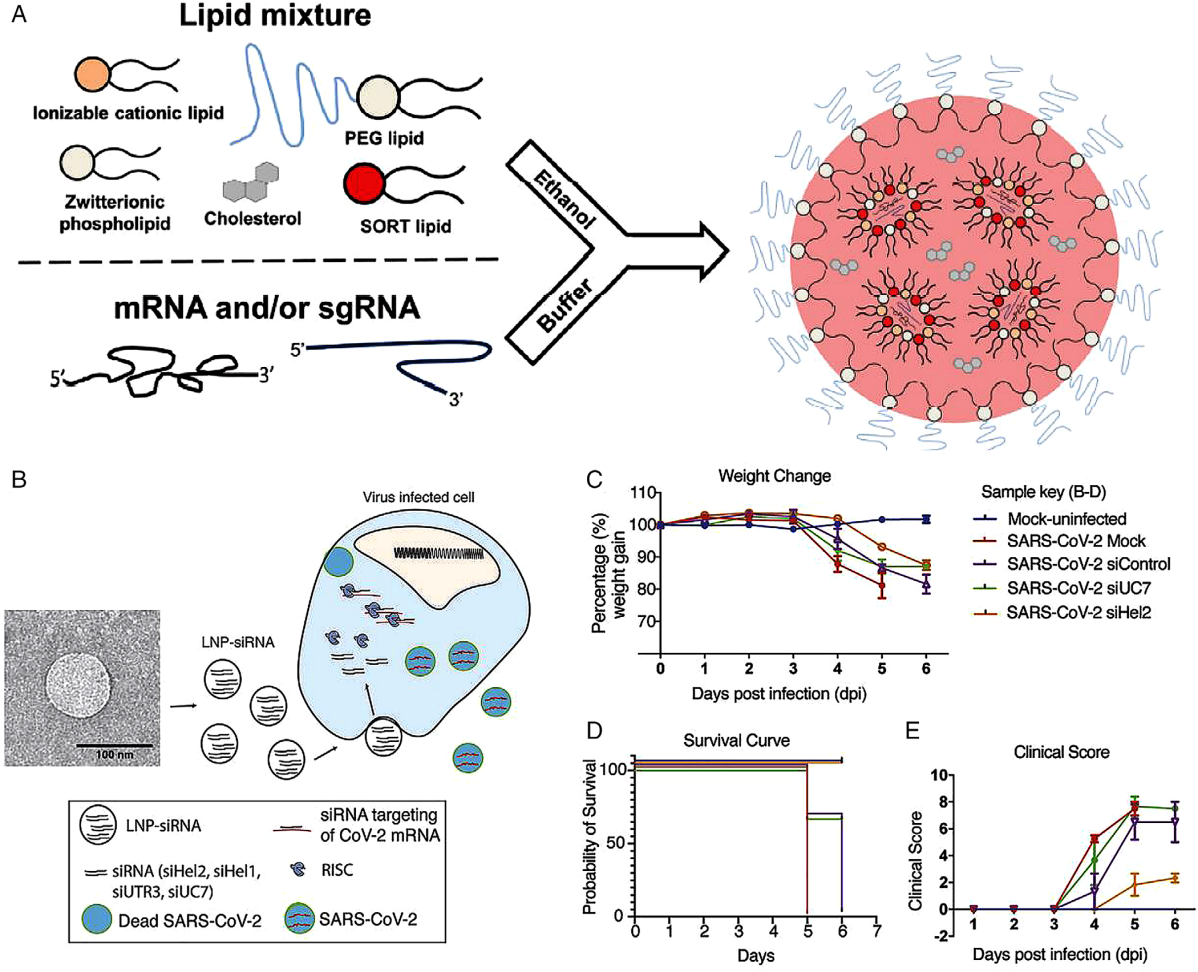
Lipid nanoparticle (LNP) systems for delivering RNA to treat COVID‐19. (A) Novel LNPs for mRNA delivery were prepared by an ethanol dilution method. The lipid mixture in ethanol and RNA in aqueous buffer were mixed rapidly and incubated at room temperature to form LNP nanoparticles. (A) Reproduced with permission.^119^ Copyright 2020, Springer Nature. (B) The transmission electron microscopy (TEM) images of LNP–siRNA and schematic of the LNP loaded with siRNA for gene therapy. (C–E) The inhibition of SARS‐CoV‐2 infectivity by LNP–siRNA in vivo. The body weight change (C), probability of survival (D), and clinical score (E) of K18‐hACE2 mice after being infected by SARS‐CoV‐2 were evaluated. (B–E) Reproduced with permission.^118^ Open access

Gene therapy by using small interfering RNA (siRNA) is a potentially promising strategy to inhibit the replication of RNA viruses. However, siRNA is unstable and negatively charged. It can be easily decomposed in the physiological environment. To overcome these restrictions, various nanomaterials have been developed to deliver siRNA in vivo, such as dendrimers, polymers, lipids, iron oxide nanoparticles, silica, and gold nanoparticles.^115^, ^116^, ^117^ Recently, three siRNAs against SARS‐CoV‐2 have been identified through the screening.^118^ Simultaneously, two novel lipid nanoparticle delivery systems have also been developed to deliver these siRNAs to the lungs and other organs (Figure 12A,B). Through intravenous injection of lipid nanoparticles encapsulating of siRNA into mice, the replication of viruses was robustly repressed in the lung, delaying the onset of COVID‐19 symptoms (Figure 12C–E).^118^

## 8 PERSPECTIVES AND CURRENT CHALLENGES

Up to now, the rapid spreading of COVID‐19 has continued for over than one and a half years. It seems that this virus will likely survive with humans for a period of time. Despite the fact that massive scientific effort has been taken to understand SARS‐CoV‐2, we know less about this virus than we would like to know. The biology and virus‐host interaction of SARS‐CoV‐2 remain largely unclear. Thereby, numerous efforts should be underway to study the virological characteristics of SARS‐CoV‐2, which can realize the advent of novel diagnostic, therapeutic, and preventive strategies against COVID‐19 with high effect. Additionally, some SARS‐CoV‐2 mutants with stronger transmission ability have appeared and caused a severer threat to global health. Facing this challenge, we should guarantee the continuous monitor of SARS‐CoV‐2 at the genomic level to promptly identify any phenotypic changes of the virus, which may challenge the efficacies of current drugs and vaccines. Moreover, a database containing genomic sequence information of coronaviruses should be developed. This database can trace the cross‐species transmission route and nature origin of SARS‐CoV‐2 and other potential novel human‐infecting coronaviruses. In a word, COVID‐19 is one unprecedented challenge for all the human beings. Tackling this challenge requires the long‐term effort of every individual.

On the other hand, nanobiotechnologists have made their own strides for the challenge. Actually, the development of nanobiotechnology in the field of the antiviral research has brought us powerful weapons against COVID‐19.^120^, ^121^ A series of nanomaterial‐based biosensors were designed to get faster, more sensitive and accurate results of viral detection.^107^, ^108^ Printed polystyrene nanochains assays modified with specific antibodies could realize the colorimetric quantification of the virus just by a portable smartphone.^122^ The surfaced‐enhanced Raman scattering (SERS) biosensor based on ACE2‐engineered gold nanostructures could even detect the SARS‐CoV‐2 at the single‐virus level.^123^ Lipid nanoparticles (LNPs) have already been used maturely and they can make mRNA vaccines more stable for in vivo delivery.^61^, ^62^ Lipids contain positively charged groups can self‐assemble with negatively charged mRNA molecules to form nanoparticles and promote the delivery of mRNA during cellular uptake.^110^, ^119^ More and more novel LNP systems are on the way to be used for delivering vaccines or other COVID‐19 drugs (Figures 10 and 12).

In addition, nanoparticle vaccines based on self‐assembly of natural protein produced a stronger immune response than mRNA vaccines.^124^, ^125^ Given their extremely high safety, nanoparticle vaccines hold great promise for preventing COVID‐19. Cellular nanosponges composed of polymer cores and cell membranes could simulate host cells, attract, and neutralize SARS‐CoV‐2 through natural cell receptors, thereby generating a broad‐spectrum antiviral strategy.^121^ The further increasing of the surface heparin concentration on the cellular nanosponges could promote its inhibitory effect on SARS‐CoV‐2.^126^ As the vector of antiviral drugs, nanomaterials cannot only control the release of drugs, but also decrease the side effects by targeted delivery.^117^, ^118^

We can see the successes of nanotechnology‐based strategy from detecting, preventing to treating the SARS‐CoV‐2. However, some limitations of the current nanotechnology are significantly existing in objective reality. A lot of works based on nanotechnology including anti‐viral nanomaterials and vaccines are far from the actual application. It is usually a long time due to the strict safety and effectiveness evaluation before the public use is approved. More practical design strategy should be considered by scientists for meeting the sustained outbreak and fast spread of SARS‐CoV‐2. In addition, the potential side‐effects and toxicity of some metal and carbon nanomaterials on health are worrying and noteworthy.^127^, ^128^ More biocompatible and advanced nanobiotechnologies using biodegradable biomaterials, such as biopolymers and polypeptides, should be developed to solve current problems in nanobiotechnological methods. Overall, although there are limitations now, nanobiotechnologies have laid out their opportunities for the war against COVID‐19.

## 9 CONCLUSION

In summary, we first generalized the virological properties of SARS‐CoV‐2 in this review and pointed out the viral life cycle, laying the foundation for developing various strategies against COVID‐19. Second, the viral transmissibility, as well as different symptoms and transmission pathways of COVID‐19, were described to demonstrate the rapid spreading of SARS‐CoV‐2 and the severe threat to global health. Subsequently, detailed introductions to various diagnostic, preventive, and therapeutic strategies against COVID‐19 were accompanied by the presentation of typical cases and discussion of in‐depth mechanisms. In particular, nanobiotechnology contributions in overcoming COVID‐19 were illustrated in detail, suggesting that it holds great opportunities for nanobiotechnology to be applied for the efficient diagnosis, prevention, and treatment of SARS‐CoV‐2 infections.

## CONFLICT OF INTEREST

The authors declare that there is no conflict of interest.
